# Implementing evidence-based obesity management guidelines requires development of medical competencies: A commentary outlining future directions in obesity education in Canada

**DOI:** 10.1016/j.obpill.2023.100086

**Published:** 2023-08-18

**Authors:** Taniya S. Nagpal, Nicole Pearce, Khushmol Dhaliwal, Joseph Roshan Abraham

**Affiliations:** a Faculty of Kinesiology, Sport, and Recreation, University of Alberta, Edmonton, Alberta, Canada; b Obesity Canada, Edmonton, Alberta, Canada; c Department of Family Medicine Faculty of Medicine and Dentistry, College of Health Sciences, University of Alberta, Edmonton, Alberta, Canada

**Keywords:** Education, Guidelines, Health promotion, Obesity, Patient care

## Abstract

**Background:**

This commentary provides an overview of forthcoming activities by Obesity Canada (OC) to inform obesity competencies in medical education. Competencies in medical education refer to abilities of medical professionals to appropriately provide patients the care they need. A recognized Canadian framework for informing medical competencies is CanMEDs. Additionally, the Obesity Medicine Education Collaborative (OMEC) provides 32 obesity specific medical competencies to be integrated across medical education curriculum. OC released the first globally recognized Adult Obesity Clinical Practice Guideline (CPGs) in 2020 inclusive of 80 recommendations. Referring to the CanMEDs and OMEC competencies, OC is developing medical education competencies for caring for patients who have obesity in line with the recent CPGs that can be applied to health professions education programs around the world.

**Methods:**

Activities being completed by OC’s Education Action Team include a scoping review to summarize Canadian obesity medical education interventions or programs. Next, with expert consensus a competency set is being developed by utilizing the CanMEDs Framework, OMEC and the CPGs. Following this, OC will initially survey undergraduate medical programs across the country and determine to what degree they are meeting the competencies in content delivery. These findings will lead to a national report card outlining the current state of obesity medical education in Canada within undergraduate medical education.

**Results:**

To date, OC has completed the scoping review and the competency set. The Education Action Team is in the process of developing the survey tools to assess the current delivery of obesity medical education in Canada.

**Conclusion:**

The evidenced-based report card will support advocacy to refine and enhance future educational initiatives with the overall goal of improving patient care for individuals living with obesity. The process being applied in Canada may also be applicable and modified for other regions to assess and better obesity medical education.

## 1 Introduction

Obesity is a chronic disease caused by an interplay of biological and environmental factors and is characterized by dysfunctional or excess adipose tissue that impairs health [1]. Despite global recognition of obesity as a complex and relapsing disease, adoption and implementation of evidence-based obesity clinical practice guidelines, including its refined definition that extends beyond historical use of body mass index (BMI) alone, appears to be lagging [2]. Obesity care and management is individualized and supporting medical professionals with providing patient-centred care is integral [1]. Importantly, clinical change and implementation is best adopted through educating future generations of health professionals and with continuing education opportunities; educators require guidance and direction on training prospective frontline healthcare providers on obesity management in line with up-to-date evidence [3]. This commentary advocates for development of medical education competencies for obesity management that can be integrated into future curriculum for health professionals in Canada. We also offer an outline of prospective education activities being undertaken by Obesity Canada, to present a list of medical competencies that may be integrated into future health professions education curriculum.

## 2 Obesity Canada

Obesity Canada (OC) is Canada’s largest and only registered charity dedicated towards improving the lives of Canadians living with obesity [4]. Founded in 2006, Obesity Canada currently has more than 50,000 members, of which, the majority of members are healthcare professionals. Obesity Canada’s objectives are to address and mitigate the social stigma associated with obesity and to improve the way policy makers and health professionals approach obesity, by considering patient-centred practices, offering non-judgmental and inclusive care, and increasing access to evidence-based prevention and management resources [4]. In 2020, OC co-led and published the Canadian Adult Obesity Clinical Practice Guideline [1]; this guideline is recognized internationally for being a comprehensive evidence-based resource for obesity management and one of the only obesity guidelines to have also incorporated recommendations for mitigating social challenges like weight stigma. Since the release of the guideline in Canada, several other countries have translated the document for use, such as Chile and Ireland [5,6]. The guideline is considered a modifiable tool that can be adopted for use in other countries [7]. In fact, the guideline has been endorsed by 15 national and international organizations including the United States obesity organizations who intend to adopt them [7]. Key reasons for the guideline’s worldwide recognition include the development process involved a person-centred approach with several adults living with obesity, the Grading of Recommendations, Assessment, Development and Evaluations was used to evaluate recommendations, a primary care framework was used throughout the document which offers familiarization for healthcare professionals around the world, it includes 19 chapters and 80 recommendations evincing its thoroughness, and it was informed by experts in the field who dedicated more than 6000 volunteer hours [7]. In addition, OC will soon be releasing comprehensive paediatric obesity guidelines. In addition to these guidelines, OC is committed to continuing to provide obesity education for clinicians around the world including the United States [8]. OC’s online education platform hosts an abundance of training resources for clinicians anywhere in the world to utilize [8]. As well, they produce several learning events open to global attendees such as a Connected Conversations learning series [9], a biennial research Summit [10], and many videos that can be integrated into public or academic lectures to translate research for general audiences [11]. Presently, OC is working towards developing a national obesity care framework focused on four pillars: Education, Research, Policy, and Community [4]. Each pillar includes experts spanning several areas such as education, medicine, research, advocacy and each pillar includes representation from individuals with lived experience of obesity. Each pillar is using evidence-based approaches to offer future directives for obesity care in Canada. Briefly, the research pillar is outlining necessary obesity research questions that require attention, the policy pillar is delineating an obesity prevention and management policy framework, and the community pillar aims to grow the patient community and generate public and political support for obesity care in Canada [12]. The education pillar aims to identify gaps and opportunities to enhance obesity-related curricula in current health education that is consistent with the Canadian Adult Obesity Clinical Practice Guidelines [4]. This commentary outlines the activities being undertaken by the Education Action Team within the education pillar.

## 3 Current education on obesity management and future directions

In Canada and around the world, prevalence of obesity has been increasing with current national estimates (relying primarily on measures of BMI) indicating approximately 25% of adults are living with obesity [13]. National direct medical costs for obesity (including hospital admission, medication, physician fees, and emergency room visits) is approximately $3.9 billion [13], and this underscores that obesity care needs to be addressed by many medical professionals spanning various specializations. Despite the increasing prevalence of obesity and its strong association with comorbidities and complications resulting from the disease itself, health professionals lack practical tools to address patient concerns, including a paucity of training on behaviour-change approaches, strategies to prevent weight bias and stigma, and comprehensive care options for multifactorial causes [14,15]. In fact, a recent Canadian post-secondary institution was surveyed on obesity perspectives and identified that most undergraduate students relied only on physical inactivity and poor nutrition as causal factors and management approaches for obesity [16]. Although healthy behaviours like physical activity and nutrition are integral to obesity care [1], alluding that they are the only determinants of obesity ignores the abundance of research that points out that obesity is caused and managed by an interplay of several physical, mental and environmental factors that all may need to be addressed in collaboration with healthy behaviour change as well [1]. Numerous cross-sectional investigations in North America that have aimed to summarize overall knowledge and confidence among practicing healthcare providers for obesity management have consistently found poor self-efficacy, lack of obesity-specific training, provider discomfort with having weight-related discussions, and a desire for further education to improve quality of care for patients living with obesity [[17], [18], [19], [20], [21]]. Accordingly, these findings may speak to the need to integrate better understanding in education on the diverse causal factors of obesity and approaches to care. Insufficient obesity education and training can result in patients receiving delayed care for obesity and comorbidities, poor patient-provider rapport, and health inequities [22].

With medical education in particular, North American undergraduate medical schools incorporate little training in evidence-based obesity management [20,23]. For example, previous studies exploring obesity-related medical education in US and Canadian family medicine clerkship programs noted that obesity was most commonly taught in curricula in the context of obesity-related complications (e.g., diabetes), or during discussion on lifestyle behavioural changes such as modifications in diet and exercise [23]. Current research and the Canadian Obesity Adult Management Guidelines put an emphasis on the biological and genetic aspects of obesity, and on mitigating bias and discrimination in obesity medicine [1]. Moreover, current guidelines offer obesity measurement tools, such as the Edmonton Obesity Staging System, to measure obesity and its risk beyond reliance on BMI [1].

**Fig. 1:**
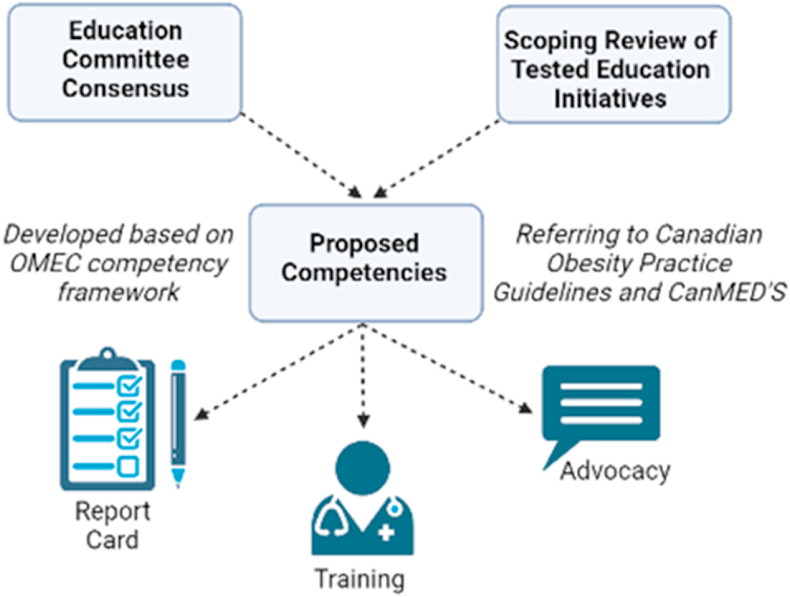
Visual representation of activities to develop obesity competencies and future directions. This figure was created using biorender.com Activity 1 is exemplified as the Scoping Review Activities 2, 3 and 4 are exemplified as the Education Committee Consensus Activity 5 is exemplified as the report card Training and Advocacy are other future outcomes that can occur in Canada and around the world using our developed competencies OMEC - Obesity Medicine Education Collaborative CanMEDs - Canadian Medical Education Directions for Specialists.

To inform Canadian medical education curricula to improve obesity-related content and competencies, the proposed objectives are currently being undertaken by Obesity Canada’s Education Team inclusive of documenting current educational practices in Canada, identifying critical gaps, and developing a comprehensive competency set for medical education that encompass undergraduate, postgraduate, and continuing professional development that can be integrated into future medical curriculum and across health professions education programs around the world. The Education Action Team is multidisciplinary including obesity researchers from both basic and applied sciences, policy and advocacy specialists, patients living with obesity, experts in medical education, and health professionals from various disciplines (e.g., behavioural psychology, family physicians, endocrinology). Findings from this work have the potential to enhance obesity medical education in Canada, as well as having global relevance. In particular, the approach being used to develop obesity medical education competencies and assess the current state of obesity education in Canada (outlined below), can be modified and applied to other regions and health professions education programs around the world:

Summary of Activities being Undertaken (Fig. 1).

1. A scoping review on published literature on obesity related educational initiatives or interventions delivered in Canadian health-related post-secondary programs.
2. Development of Canadian obesity education competencies and milestones which can be utilized across the educational spectrum.
3. Development of nested Entrustable Professional Activities (EPAs) from the obesity education competencies for assessing important tasks in the clinical setting.
4. Survey of medical schools in Canada to see which competencies, milestones and EPAs may already be addressed or not addressed in existing curriculum.
5. Compile the survey data, design and disseminate an obesity education report card that presents the findings of this project to Canadian obesity stakeholders, policy makers, educators and the public.

A scoping review in line with the Preferred Reporting Items for Systematic Reviews - Scoping Reviews extension will be conducted to achieve Activity 1 [24]. A scoping review seeks to summarize thematically or narratively findings pertaining to an emerging topic with the goal of outlining critical gaps and future directions [24]. A scoping review tends to serve broad topic areas that would benefit from a thematic summary as opposed to a systematic review that may be more specific with extensive research done on the topic already [25]. Accordingly, the review is a ‘scoping’ exercise to map what is known on the selected topic [25]. The scoping review will provide a summary of previous obesity educational interventions that have been implemented and tested in health programs in Canada and offer recommendations for future research in obesity medical education. Therefore, as our main objective of the scoping review is to map what already exists on this topic in Canada and to provide future directives, scoping review methods were deemed appropriate. We will search research databases (e.g., PubMed), as well as grey literature through dissertations for primary articles on this topic. Notably, limitations of scoping reviews include a lack of quality assessment of the included studies [25]. However, we do not anticipate a large volume of included studies and the main goal is to summarize attempted educational initiatives on obesity to date and therefore quality assessment would not be a priority yet, but this is certainly an important future direction as more obesity medical education interventions are developed.

Activity 2 will be achieved by consulting with diverse expertise including clinicians, educators, researchers, and persons with lived experience of obesity. Using the Obesity Medicine Education Collaborative (OMEC) competencies and the Canadian Medical Education Directions for Specialists (CanMEDS) competency framework, we will develop Canadian obesity education competencies for medical education that can be applied to health professions programs [3]. Medical education competencies refer to essential abilities of a physician to care for patients [3]. The OMEC competencies were developed in 2019 to inform curriculum and assessment for undergraduate and postgraduate medical education programs, although they could also be adapted to other health programs such as for nurse practitioners and physician assistants [3]. The impetus for these competencies could be seen in multiple areas, including a broad gap in obesity education in the United States as highlighted by a report in 2007 from the Association of American Medical Colleges, a comprehensive review of the medical licensing exams, and numerous papers voicing concerns about the lack of adequate training around obesity care in the country [26]. In response to this, the OMEC provided 32 core competencies [3]. Building from this, we will develop Canadian obesity education competencies that align with the Canadian Obesity Clinical Practice Guidelines. A framework to guide Canadian competencies is the CanMEDs [1,27]. Not only is CanMEDs the most widely used physician competency framework in the world, it also offers a uniquely Canadian perspective for curriculum and assessment through competencies that define essential abilities for all areas of medical education and practice in Canada. CanMEDS was developed by the Royal College of Physicians and Surgeons of Canada for specialist physicians and was formally approved in 1996 [28]. The framework now represents the necessary competencies for all areas of medical practice and provides a foundation for medical education and practice in Canada, no matter the specialty [29]. It was created to address rapidly evolving changes and trends in medical education in the 1990s along with a rise in the demand from the public for accountability in the profession [29]. CanMEDs also offer a framework that can be integrated into other health professions’ education curriculum. Adherence to the developed competencies would ultimately allow for identification of qualified health professionals for obesity management in Canada [28,29].

Once the obesity competencies have been developed, the Education Action Team along with expert consultants will then identify key milestones and EPAs will be established (Activity 3). Milestones refer to educational statements that show how competence is expected to progress over the course of their career from novice to mastery [28]. For example, a competency may be *“Perform a patient-centred clinical assessment and establish a management plan”*, and corresponding milestones could be “*Identify the concerns and goals of the patient and family for the encounter; or prioritize which issues need to be addressed during future visits or with other health care practitioners”*. As seen in the example, competencies and milestones are largely theoretical and do not indicate what actions learners need to take to achieve them. To fill this gap, EPAs were created as a unique form of clinical assessment, as they represented the evaluation of tasks that learners are expected to attempt at completing once they have been felt to have obtained the necessary competence by their preceptors in several key competency areas designated by a health professions program [28]. In order to move to further stages of training, learners would need to complete the appropriate number of EPAs designated by the program. Nested EPAs are for specialty organizations, in this case OC, and can easily fit into an existing medical education program’s EPA structure as they represent specialty-specific competencies that can be assessed on the trajectory towards completing the larger EPA [28]. An example of this would be a nested EPA on taking an obesity-focused clinical assessment that would fit into the broader EPA requirements for a patient-centred clinical assessment in a particular health professions program. By using the CanMEDS competencies, obesity specific competencies can be easily nested into existing assessment structures in various health professions education programs. In summary, by the end of Activity 3 we will have an obesity competency set and corresponding milestones and nested EPAs informed by expert consensus with reference to the OMEC competenciesm the CanMEDS competency framework, and the Canadian Obesity Clinical Practice Guidelines. An important note, the produced list of competencies will serve as guidance for medical professionals. A key component of the 2020 Canadian Adult Obesity Management guideline was the emphasis on patient-centred care for obesity. The goal of these competencies is to try to best prepare medical professionals with the skills and confidence to offer patient-centred care. The milestones and EPAs serve as guiding directives to achieve the competencies. To best equip prospective medical professionals with the necessary knowledge, tools, and skills to develop the competencies we need to provide medical training that supports the establishment of the competencies which is the motivation for Activities 4 and 5.

To complete Activity 4, Canadian medical school officials will be surveyed to identify which of the competencies crafted by OC are already being covered and the degree to which they are covered (e.g., inclusion in modules, total hours spent). We will design a survey tool that will be completed by Canadian medical school officials. The survey will include the obesity education competency and associated milestones and current obesity medical curricula will be evaluated against these to understand what is already being taught to prospective medical professionals and what areas are lacking. Of note, although this is all being done in a Canadian context, non-Canadian clinicians and medical educators can still review our developed list of competencies, milestones and EPAs to understand what is required of a medical professional to sufficiently care for patients who have obesity. Moreover, our process of developing the competencies and surveying medical schools on what competencies are being met could be modified and used in other countries as well to capture the landscape of obesity education in that area.

Based on the findings of the survey with medical schools, we will develop a report card on the current state of obesity-related education in Canada (Activity 5). A report card provides an overview of a particular system or program [30]. The summarized format of report cards makes them particularly useful tools for informing policy makers and guiding advocacy efforts, by highlighting the areas that need improvement [30]. A further advantage of report cards is that they can assist in making comparisons over time through easily replicated evaluation methods [30]. Therefore, the final product as a result of this project will be a synthesized report card that can be used to advance future obesity-related medical education curricula in Canada. Moreover, the guiding steps and the final list of competencies may be adopted and adapted by other countries and health authorities to critically evaluate their current state of obesity management education and inform improvements. Following the report card, a future objective will be to outline implementation strategies to integrate the competencies into curriculum and assessment and have a plan for quality assurance, with nested EPAs representing the cornerstone for assessment of the competencies in clinical practice settings.

## 4 Impact

Through this initiative, OC will have a comprehensive understanding of the current national state of health education pertaining to obesity, and clear directives on how to improve curriculum content. The scoping review will identify what has been implemented or tested in terms of obesity education in Canada and help shape future directions. The development of competencies and milestones, followed by surveying health programs will further explore existing obesity content especially in medical education in Canada. The report card will provide a comprehensive overview on the current state of affairs across the country on the availability of obesity-related education in medicine and importantly, offer clear recommendations to enhance curriculum and steps to assess knowledge among health professional groups around the world.

The importance of equipping prospective health professionals with a comprehensive understanding of obesity etiology, management techniques, and patient experience, is to improve their delivery of high-quality care that is evidence-based, person-oriented, and free from biases. Overall, advancing obesity education will improve healthcare provider knowledge and delivery of comprehensive person-oriented care for patients living with obesity.

## 5 Conclusion

Medical education competencies refer to critical skills and abilities of medical professionals to provide appropriate patient care and facilitate quality health outcomes. Establishing medical competencies is an important step towards improving quality of care for patients living with obesity. An existing list of core competencies for obesity is provided by the OMEC. Building from this, Obesity Canada has taken the initiative to create Canadian obesity medical competencies that are in line with the Canadian Obesity Clinical Practice Guidelines. By referring to the CanMEDs framework, which is a guiding framework on developing medical competencies for the practice of medicine, Obesity Canada’s Education Action Team is pursuing a series of activities outlined in this commentary. The activities include a scoping review on already existing and tested obesity medical education interventions in Canada, and expert consensus establishing obesity medical competencies, milestones and EPAs. Based on the competencies, medical schools will be surveyed to assess which areas may already be addressed in existing curriculum and which are lacking, leading to a report card that will summarize the current state of obesity medical education in the country. The report card will serve as an evidence-based tool that can support advocacy for refining obesity medical training.

## Authorship statement

NP and JRA led the development of the projects outlined in this commentary. TSN, NP, KD, JRA are all members of the Education Action Team with Obesity Canada and are leading the proposed activities outlined in the commentary. TSN drafted the commentary, and all authors reviewed and edited. All authors approved the final submission.

## Ethical review

As this is a commentary, ethical review was not required.

## Funding

This research was funded by Obesity Canada's Fund for Obesity Collaboration and Unified Strategies (FOCUS) initiative in addition to in-kind support from the scientific and professional volunteers engaged in the process.

## Declaration of Artificial Intelligence (AI) and AI-assisted technologies in the writing process

During the preparation of this work the authors did not use AI-assisted technologies.

## Declaration of competing interest

The authors declare no conflict of interest. Nicole Pearce is an employee of Obesity Canada. All are volunteer members of Obesity Canada’s Education Committee.
